# Profile of individuals with low back pain and factors defining chronicity of pain: a population-based study in Ethiopia

**DOI:** 10.1007/s11136-022-03148-5

**Published:** 2022-05-14

**Authors:** Getahun Kebede Beyera, Jane O’Brien, Steven Campbell

**Affiliations:** School of Nursing, College of Health and Medicine, University of Tasmania, Launceston, TAS Australia

**Keywords:** Associated factors, Chronicity of pain, Low back pain, Population-based study, Profile

## Abstract

**Purpose:**

Low back pain (LBP) is the most prevalent public health problem globally, second only to headaches in the ranking of painful disorders that affect human beings. However, evidence about the profile of LBP patients is lacking in low-income countries for appropriate management approaches. This study examined the profile of individuals with LBP and factors defining chronicity of pain in Ethiopia.

**Methods:**

A population-based cross-sectional study design was used to collect data from 1812 adults (≥ 18 years) with LBP at present. Data were collected by interviewing the study participants using an instrument developed and validated in the same study population. The instrument includes socio-demographic information, health behaviours/lifestyle habits, beliefs about pain, and pain and general health-related characteristics of the participants. Data analysis was performed using R version 3.5.1. Both unconditional and conditional logistic regression models were fitted and Odds Ratio (OR) with 95% confidence intervals (95% CIs) were computed to identify factors significantly associated with chronicity of pain at *p* ≤ 0.05 significance level.

**Results:**

Negative beliefs about pain, a varying degree of pain interference with daily and social activities, complaining of pain in other anatomical sites other than the low back region, general health status rated as *not excellent*, depressive symptomology, and sleeping problems/insomnia were common within the profile of individuals with LBP. Age, educational level, residential setting, beliefs about pain, and depressive symptomology were found to have a statistically significant association with chronicity of pain.

**Conclusions:**

This study provides an overview of the profile of individuals with LBP and factors defining chronicity of pain, assisting clinicians to design appropriate management strategies to improve patients' outcomes.

**Supplementary Information:**

The online version contains supplementary material available at 10.1007/s11136-022-03148-5.

## Introduction

Low back pain (LBP) is pain localised below the line of the 12th rib and above the inferior gluteal folds, with or without leg pain [1, 2]. LBP is the most prevalent public health problem globally, second only to headaches in the ranking of painful disorders that affect human beings [3]. The pain can be either specific or non-specific, based on causal factors. Specific LBP is caused by known pathology, such as infection, osteoporosis, rheumatoid arthritis, fracture, or tumour [4], whereas non-specific LBP is not attributable to a recognisable, known specific pathology [4–6]. LBP can affect up to 80% of all individuals [7], and non-specific LBP shares approximately 90% of it [4, 5]. The most recent epidemiological data demonstrate that the annual global incidence of LBP is 245.9 million with an overall prevalence of 577 million and disability-adjust life years (DALYs) accounting for 64.9 million [8]. Evidence shows that an individual with LBP at a point in time will have a strong tendency towards having continuing pain or having it again [9]. Indeed, it has been proven to be a chronic disease [10], marked by a stable pattern of episodes, which may occur frequently or infrequently and these episodes may be of short or of long duration [11].

Studies have shown that a large proportion of people with chronic LBP also report mental health disturbances, such as elevated depressive and anxiety symptoms, as well as disability in activities of daily life[12–14]. These effects, in turn, are seen to worsen the experience of pain [13], suggesting complex relationships between pain, pain outcomes, and related quality of life. When measured using the *36-Item Short Form Survey (SF-36)*, an association between lower summary score (poor health-related quality of life) and LBP was seen [15], particularly among individuals with chronic LBP [2]. Socio-demographic variables such as older age [16, 17], and health behaviours/lifestyle habits such as obesity and lack of physical exercise [16] have also been reported as factors associated with increasing occurrence of poor health-related quality of life in people with LBP. Therefore, it is clear that LBP is a complex health condition with irregular and uncertain outcomes [18], and continues to be one of the most controversial and difficult health conditions to manage for clinicians, patients, and policy makers [19]. LBP, particularly when it moves to the chronic phase, and combined with other comorbid conditions, is known to have an array of negative health impacts, with significant, life-changing psychological and social consequences [20]. Effective management of pain in its early phase could be important to minimise the substantial burden of LBP [21, 22].

In Ethiopia, infectious diseases, such as tuberculosis, malaria, diarrhoeal diseases, HIV/AIDS, and lower respiratory infections, are the leading health issues. A study undertaken by Misganaw et al. [23] found that in 2015, the all-cause crude death rate was 680.9 (95% uncertainty interval (UI): 505.1–913.9) per 100,000 population. Of this, 337 (95% UI: 273.8–421.3) per 100,000 population and 286.9 (95% UI: 188.1–423.0) per 100,000 population death rates were caused by communicable and noncommunicable diseases, respectively. The same study further showed that HIV/AIDS and tuberculosis together caused 72.8 (95% UI: 50.9–101.0) deaths per 100,000 population. Diarrhoea, lower respiratory infections, and other common infectious diseases accounted for 147.4 (95% UI: 113.9–191.0) deaths per 100,000 population, while cardiovascular diseases and neoplasms accounted for 120.5 (95% UI: 77.1–177.5) and 55.6 (95% UI: 32.8–89.8) deaths per 100,000 population, respectively. Combined with these long-standing infectious diseases and other common noncommunicable diseases, the burden of LBP in Ethiopia may be large and amount to a considerable public health concern. However, there has been no study devoted to epidemiological data about the profile of individuals with LBP in Ethiopia to date, limiting the effectiveness of management strategies for improved outcomes. This study was therefore aimed to offer a detailed epidemiological information about the profile of individuals with LBP and factors associated with chronicity of pain in Ethiopia. The findings may assist to develop sensible and practical planning for appropriate pain management strategies and improved outcomes.

## Methods

### Study design and setting

A population-based cross-sectional study design was used to describe the profile of individuals with LBP, residing in South-west Shewa zone of Oromia regional state, located in central Ethiopia.

### Sample size

A single population proportion formula was used to determine the study sample size. The calculation involved a 95% confidence level (*Zα*/2 = 1.96), and a low margin of error (*d* = 4%). The expected proportion of chronic LBP (*p* = 50%) was assumed, because of the absence of any previous study, and a design effect (DE) of 3 was also factored in considering a multi-stage sampling technique. In this way, a total of 1981 adults with LBP were included in the study with a 10% non-response rate [24].

### Sampling procedure

A multi-stage sampling technique was used to select the study participants. Firstly, the districts in South-west Shewa zone were stratified into urban and rural. Three districts (one urban and two rural) were then selected as endorsed by Cohen et al. [25]. Finally, two wards were randomly picked from each of the three selected districts using the OpenEpi Random number generator [26], totalling six *wards*. The households within the selected *wards* were then selected proportionally using a systematic random sampling method. *Important to note that while the households were the sampling units, the individuals with LBP (*≥ *18 years of age) were the study units.*

### Data collection instrument

The instrument used in this study was first developed and validated in the same study population as described elsewhere [27]. The findings of the validation showed that the instrument is both valid and reliable to measure the profile of individuals with LBP. The instrument covers items measuring socio-demographic characteristics, health behaviours/lifestyle habits, and pain and general health-related profile of individuals with LBP (Supplementary materials). Socio-demographic factors such as gender, age, ethnicity, educational level, residence, marital status, and living conditions were included in the instrument. To measure health behaviours/lifestyle habits, information about smoking (smoking status, duration of smoking, and number of cigarettes smoked per day), alcohol consumption status and frequency of consumption, and khat chewing status and frequency of chewing were collected. Beliefs about pain were measured with a five-item scale, with response options ranging from *strongly agree* to *strongly disagree*. Optimistic individuals who scored above the mean were rated as having *positive beliefs*, while those scored below the mean score were rated as having *negative beliefs*. Information about intensity of pain (measured on a 10-point Numerical Rating Scale [NRS]), duration since pain onsets, pain interference with daily and social activities, whether pain spreads down the leg(s) or not and whether it caused time off work or not, number of days off work due to pain, and whether pain present in other anatomical sites other than the low back region or not were included in the instrument to assess pain-related profile of individual participants. General health-related information such as self-rated general health status and the presence of depressive symptomology and sleeping problems/insomnia were also covered in the instrument. The specific methods used to measure and define the variables such as intensity of pain, depressive symptomology, and insomnia have been described elsewhere [24].

### Data collection

The data were collected by graduates with a First degree in health and/or a related field (*n* = 12), who were familiar with the culture, norms, and language of the local communities, but who did not know the families in the study area. For each *ward*, two data collectors were assigned, and collected the data by interviewing individuals with LBP using the Oromo language version of the instrument. In identifying cases with LBP, the individuals were directed to a picture of a person with a shaded area defining the low back region and were asked whether they had pain lasting more than one day in that region. Individuals were defined as having LBP and invited to be included in the study if they reported that they had pain in the specified body region. Only one adult with LBP was interviewed from each of the selected households. Whenever two or more eligible respondents were found in the selected household, only one respondent was chosen by the lottery method. With this method, each of the individuals with LBP was assigned a unique number, which was placed in a bowl, from which only one of the numbers was drawn at random. Where there was no eligible interviewee in the selected household, the next household was visited.

### Statistical analysis

The data were analysed using R version 3.5.1. To provide insight into the profile of individuals with LBP, descriptive analyses were carried out and the findings were presented as frequency, percentage, median interquartile range (IQR), table, and graph. Defining chronic LBP as pain lasting for more than 3-month [28, 29], both unconditional and conditional logistic regression models were also fitted and Odds Ratio (OR) with 95% confidence intervals (95% CIs) were computed to identify factors statistically associated with chronicity of LBP at *p* ≤ 0.05 significance level. The predicting factors included in the models were gender, age, educational level, residence, marital status, living conditions, smoking, alcohol consumption, khat chewing, beliefs about pain, pain interference with daily and social activities, intensity of pain, the presence of pain in other anatomical sites other than the low back region, depressive symptomatology, sleeping problems/insomnia, and self-rated general health status. Chronicity of pain was the only dependent variable included in the models.

The association between each predicting factor and chronicity of pain was first assessed independently using an unconditional logistic regression model. However, this model could not account for the effects of confounding factors and yields only crude OR with 95% CIs and *p* values. For this reason, conditional logistic regression model, where each predicting factor was matched with age and depressive symptomology, was then fitted to determine AOR with 95% CIs and *p* values. This helped to identify the true association between each predicting factor and chronicity of pain.

## Results

### Socio-demographic profile

Of the total 1981 approached individuals with a self-reported history of LBP, 1812 participated in this study, making a response rate of 91.5%. The proportion of males was slightly higher than females (984 [54.3%] vs. 828 [45.7%]). The median (IQR) of participants' age was 38 (30–50) years. The details of socio-demographic characteristics of the study participants are presented elsewhere [24].

### Health behaviours/lifestyle habits and beliefs about pain

Unhealthy behaviours such as substance use (smoking, alcohol consumption, and khat chewing) were uncommon. For example, only 58 (3.2%) of individuals with LBP were smokers with median (IQR) years of smoking 5.5 (2.75–10). Similarly, only 154 (8.5%) were khat chewers at the time of data collection. About 47% of the participants had negative beliefs about pain (Table 1).

**Table 1:** Health behaviours/lifestyle habits of individuals with LBP and their beliefs about pain (*n* = 1812)

Characteristics	*n*	%	Characteristics	*n*	%
*Smoking status*			*Khat chewing status*		
Current smoker	58	3.2	Current chewer	154	8.5
Former smoker	84	4.6	Former chewer	79	4.4
Never smoked	1670	92.2	Never chewed	1579	87.1
*Smoking duration, in years* (*n* = 58)^§^	5.5 (2.75–10)		*Frequency of chewing* (*n* = 154)		
*Number of cigarettes smoked per day* (*n* = 58)^§^	4 (3–5)		Occasionally	73	47.4
			Often	39	25.3
*Alcohol consumption status*			Always	42	27.3
			*Beliefs about pain*		
Current consumer	523	28.9	Negative beliefs	856	47.2
Former consumer	251	13.8	Positive beliefs	956	52.8
Never consumed	1038	57.3			
*Alcohol consumption frequency* (*n* = 523)					
On regular bases	101	19.3			
Occasionally	422	80.7			

^§^Median (interquartile range)

### Pain-related profile

Apart from 412 (22.7%), the remaining 1400 (77.3%) participants reported a varying degree of pain that interfered with their daily activities, ranging from *a little bit* to *very much*. In 542 (29.9%) people, time off work caused by pain was identified. Table 2 presents the details of pain-related characteristics of individuals with LBP.

**Table 2:** Pain-related profile of individuals with LBP (*n* = 1812)

Characteristics	*n*	%	Characteristics	*n*	%
*Duration since pain onsets*			*Pain interferes with social activities*		
Less than 1-month	150	8.3			
1–3 months	223	12.3	Not at all	417	23.0
Greater than 3-month, but less than 1-year	627	34.6	A little bit	400	22.1
			Somewhat	518	28.6
1–5 years	571	31.5	Quite a bit	356	19.6
More than 5-year	241	13.3	Very much	121	6.7
*Pain spread down the leg(s)*			*Days off work due to pain in the past year*		
No	1651	91.1			
Yes	161	8.9	No	1270	70.1
*Pain interferes with daily activities*			Yes	542	29.9
			*Number of days off work due to pain* (*n* = 542)^§^	8 (5–21)	
Not at all	412	22.7			
A little bit	368	20.3	*Intensity of pain*		
Somewhat	545	30.1	Mild	1053	58.1
Quite a bit	364	20.1	Moderate	356	19.7
Very much	123	6.8	Severe	403	22.2
			*Presence of pain in other anatomical sites other than the low back region*		
			No	1349	74.4
			Yes	463	25.6

^§^Median (interquartile range)

**Fig. 1:**
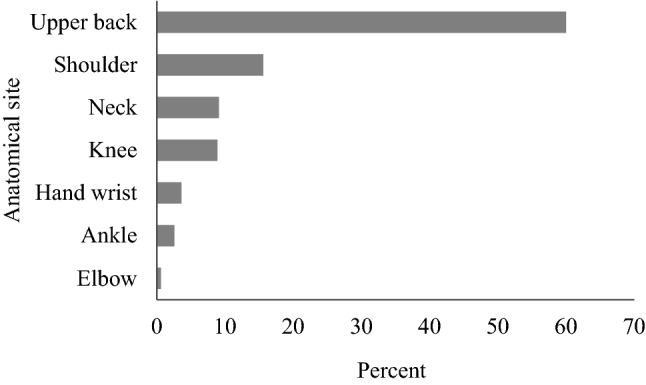
Pain in other anatomical sites other than the low back region

Pain in the upper back region was a common attendant of LBP. Of 463 (25.6%) complaints of pain in other anatomical sites other than the low back region, the majority, 278 (60%) reported pain in the upper back region, while 72 (15.6%) indicated pain in the shoulder area (Fig. 1).

### General health-related profile

More than a quarter (25.7%) of individuals with LBP indicated that they had sleeping problems/insomnia. Self-reported general health status of *fair* and *poor* were reported by 307 (17%) and 66 (3.6%) of the participants, respectively (Table 3).

**Table 3:** General health-related profile of individuals with LBP (*n* = 1812)

Characteristics	*n*	%	Characteristics	*n*	%
*Self-rated health status*			*Depressive symptomology*		
Excellent	198	10.9	Normal	968	53.4
Very good	637	35.2	Borderline case	602	33.2
Good	604	33.3	Case	242	13.4
Fair	307	17	*Had sleeping problems/insomnia*		
Poor	66	3.6	No	1347	74.3
			Yes	465	25.7

### Factors associated with chronicity of pain

In the unconditional logistic regression model, socio-demographic factors (such as age, educational level, marital status, residence, and living conditions), behavioural factors (such as alcohol consumption status and beliefs about pain), and clinical factors (such as depressive symptomology) were found to be associated with chronicity of LBP. However, when adjusting for age and depressive symptomology in the conditional logistic regression model, only age, educational status, residence, beliefs about pain, and depressive symptomology remained as having a statistically significant association with chronicity of LBP.

Increasing age group from 18–29 years was associated with increasing odds of having chronic LBP (30–39 years, AOR = 1.77, 95% CI 1.33–2.35; 40–49 years, AOR = 3.12, 95% CI 2.21–4.46; ≥ 50 years, AOR = 6.84, 95% CI 4.64–10.32). Alternatively, when age was fitted in the unconditional logistic regression model as a continuous variable, for each one-year increase in age, chronicity of LBP also observed to increase by 1.06-factor (AOR = 1.06, 95% CI 1.04 – 1.07, and *p* < 0.001). When compared with individuals who did not attend formal education, those who attended grade 1–8, grade 9–12, and those who graduated technical/vocational certificate, diploma, and degree or higher were found to have a higher prevalence of chronic LBP. Thus, those who attended grade 1–8 (AOR = 1.91, 95% CI 1.29–2.81) and grade 9–12 (AOR = 3.00, 95% CI 1.91–4.73) were 1.91 and 3 times more likely to have chronic LBP than those who did not attend formal education, respectively. Similarly, graduates of technical/vocational certificate (AOR = 2.35, 95% CI 1.36–4.11), diploma (AOR = 2.67, 95% CI 1.63–4.41), and degree or higher (AOR = 3.16, 95% CI 1.85–5.51) were 2.35, 2.67, and 3.16 times more likely to report a history of chronic LBP than their counterparts, respectively. In rural residents, the odds of reporting chronic LBP were 65% lower compared with their urban counterparts (AOR = 0.35, 95% CI 0.27–0.46).

There was also a statistically significant association between individuals' beliefs about pain and chronicity of pain. Those who had positive beliefs about pain were 33% less likely to have chronic LBP than individuals who had negative beliefs of pain (AOR = 0.67, 95% CI 0.52–0.85). In addition, a statistically significant association was observed between depressive symptomology and chronicity of pain. Compared with individuals who had no depressive symptomology, those who were at the borderline of depressive symptomology were 2.23 time more likely to have chronic LBP (AOR = 2.23, 95% CI 1.69–2.97), while those who had depressive symptomology were 1.95 times more likely (AOR = 1.96, 95% CI 1.33–2.96) (Table 4).

**Table 4:** Factors associated with chronicity of LBP

Factors	OR (95% CI)	*p*-value	AOR^‡^ (95% CI)	*p*-value
*Gender (male*^†^*)*				
Female	0.88 (0.70–1.10)	0.265	0.96 (0.76–1.23)	0.760
*Age (18–29*^*†*^*)*				
30–39	1.70 (1.29–2.25)	< 0.001	1.77 (1.33–2.35)	< 0.001
40–49	3.07 (2.18–4.37)	< 0.001	3.12 (2.21–4.46)	< 0.001
> 50	6.91 (4.71–10.39)	< 0.001	6.84 (4.64–10.32)	< 0.001
*Educational level (No formal education*^*†*^*)*				
Elementary (grade 1–8)	0.96 (0.68–1.35)	0.839	1.91 (1.29–2.81)	0.001
Secondary (grade 9–12)	0.88 (0.60–1.28)	0.494	3.00 (1.91–4.73)	< 0.001
Technical/vocational certificate	0.69 (0.42–1.13)	0.136	2.35 (1.36–4.11)	0.002
Diploma	1.09 (0.70–1.70)	0.712	2.67 (1.63–4.41)	< 0.001
First degree or higher	1.44 (0.89–2.40)	0.147	3.16 (1.85–5.51)	< 0.001
*Residence (urban*^*†*^*)*				
Rural	0.41 (0.32–0.52)	< 0.001	0.35 (0.27–0.46)	< 0.001
*Marital status (single*^*†*^*)*				
Married	2.46 (1.87–3.23)	< 0.001	1.34 (0.97–1.85)	0.077
Cohabited	0.90 (0.47–1.76)	0.750	0.81 (0.41–1.62)	0.536
Separated	1.90 (0.94–4.17)	0.087	0.95 (0.45–2.17)	0.901
Divorced	1.69 (0.96–3.13)	0.080	0.75 (0.40–1.48)	0.396
Widowed	5.04 (2.71–10.29)	< 0.001	1.15 (0.56–2.52)	0.712
*Living with (nuclear family*^*†*^*)*				
Non-nuclear family	0.51 (0.31–0.86)	0.009	0.90 (0.53–1.54)	0.686
Alone	0.62 (0.45–0.88)	0.006	0.99 (0.69–1.43)	0.951
*Smoking status (current smoker*^†^*)*				
Former smoker	1.23 (0.53–2.79)	0.625	0.89 (0.37–2.10)	0.784
Never smoked	1.11 (0.57–2.03)	0.737	1.20 (0.60–2.27)	0.584
*Alcohol consumption status (current consumer*^†^*)*				
Former consumer	1.92 (1.25–3.01)	0.003	1.55 (0.99–2.46)	0.059
Never consumed	0.88 (0.69–1.15)	0.370	1.01 (0.76–1.32)	0.963
*Khat chewing status (Current chewer*^†^*)*				
Former chewer	0.80 (0.42–1.54)	0.491	0.55 (0.28–1.10)	0.086
Never chewed	0.98 (0.64–1.46)	0.924	0.73 (0.47–1.11)	0.155
*Beliefs about LBP (Negative beliefs*^†^*)*				
Positive beliefs	0.72 (0.57–0.90)	0.005	0.67 (0.52–0.85)	0.001
*Depressive symptomology (Normal*^†^*)*				
Borderline case	2.24 (1.71–2.95)	< 0.001	2.23 (1.69–2.97)	< 0.001
Case	2.12 (1.46–3.15)	< 0.001	1.96 (1.33–2.96)	< 0.001

*OR* odds ratio (crude), *AOR* adjusted odds ratio

^‡^Adjusted for age and depressive symptomology (*Note:* In the model to compute AOR for age and depressive symptomology, both variables were adjusted for each other); *CI* confidence interval

^†^Reference category

## Discussion

This study aimed to offer the first epidemiological information about the profile of individuals with LBP and factors defining chronicity of pain using a representative sample of individuals with LBP from both urban and rural settings in Ethiopia. The study identified socio-demographic characteristics, lifestyle habits, beliefs about pain, and pain and general health-related characteristics of these individuals. The study also identified statistically significant factors associated with chronicity of LBP. These included age, educational level, residence, beliefs about pain, and depressive symptomology.

### Profiles reflecting individuals with LBP

The most common pain-related characteristics identified in this study included pain interfering with daily and social activities to a varying degree, ranging from *a little bit* to *very much*, time off work due to pain, intensity of pain ranging from *mild* to *severe*, and comorbidity with other spinal pain. When LBP is classified as acute (lasts < 4 weeks), subacute (lasts 4–12 weeks), and chronic (lasts > 12 weeks), 79.4% of participants classified their pain as chronic. A considerable proportion of people with LBP were also found to have negative beliefs about pain (for example, believing that the pain makes everything in life worse and healthcare providers cannot do anything to assist the pain). In addition, depressive symptomology, sleeping problems/insomnia, and unfavourable general health status (i.e., general health status that was not *excellent* when self-rated) were also among the common profiles reflecting individuals with LBP. In general, these findings are comparable with the findings of a study that described the profile of patients with acute LBP seeking emergency departments in public hospitals in Brazil [30]. In that study, a significant proportion of patients with LBP were identified to have pain spreading down the leg(s), higher intensity of pain, 1-week lasting disability caused by pain, self-rated general health status of *good*, and psychological interference including stress, anxiety, and depression. The same study also showed that most patients with LBP were overweight with a low level of physical activity. However, body mass index (BMI) level and physical activity level were not presented in this study due to the items measuring them not meeting the factor extraction criteria (and were removed) when carrying out factor analysis to validate the data collection instrument. Several other studies [31–34] also reported that people with LBP complain about sleeping problems/insomnia, which is consistent with the current study. Uchmanowicz et al. [35] further described sleeping problems/insomnia as a statistically significant factor affecting quality of life in people with LBP and called for public health intervention.

### Socio-demographic factors associated with chronicity of LBP

Three socio-demographic factors, namely, age, educational level, and residential setting, were identified to have a statistically significant association with chronicity of LBP. There was a gradual and proportional increase in chronicity of pain with increasing age, which is similar to the findings of previous studies [36, 37]. Malta et al. [36] linked this dose–response association between age and chronicity of LBP with the changes in the body because of the ageing process, such as postural problems, decreased flexibility, and increased musculoskeletal degeneration, which give rise to exacerbation of pain.

Previous studies reported an inverse association between educational level and occurrence of chronic LBP [36, 38–40]. In those studies, lower educational level was found to increase occurrence of chronic LBP, and scholars argued that less schooling population often engaged in more strenuous work and physically demanding jobs, and reduced access to healthcare [36]. The findings of the present study, however, showed improved educational level as increasing the risk of chronic LBP. Regular physical exercise is demonstrated to have a significant role in the management of LBP [41, 42]. However, there is an argument that due to lack of access to transportation systems and poorer income, individuals with lower educational level may be involved in some beneficial physical activities, such as walking more commonly when compared with individuals with improved educational level [43]. Thus, the reason why people with improved educational level had a higher odds of reporting chronic LBP in this study could be associated with this argument.

A study investigated factors predicting occurrence of chronic LBP in Brazilian adults [36] found that the proportion of chronic LBP was higher in males living in rural than urban settings for unexplained reasons. An incongruent result was observed in this study, as those living in rural areas were found to have 65% lower odds of having chronic LBP than their counterparts living in urban areas. In a previous paper [24], it was reported that the proportion ratio of healthcare utilisation for optimal management of LBP was higher in rural than urban populations (adjusted proportion ratio = 1.69, 95% CI 1.44–1.99). Thus, the lower odds of chronic LBP in the rural population may be linked with the higher rate of healthcare utilisation in the rural population, which improves their pain conditions and halts the progression of pain to the chronic phase.

There is evidence that individuals with unhealthy lifestyles, such as smoking cigarettes, have a higher predisposition to develop chronic pain, because nicotine could lead to an activation of the immune system and predispose people with a history of smoking to LBP, rheumatic diseases, and other health conditions [39, 44]. In terms of chronicity of pain, evidence is lacking to demonstrate the association between unhealthy lifestyles and LBP. The findings of the current study also did not find a statistically significant association between chronicity of LBP and unhealthy lifestyles, including smoking and chewing khat, except that alcohol intake was marginally associated increased chronicity of pain.

### Psychological factors associated with chronicity of LBP

According to Teixeira et al. [45], patients' and healthcare providers' negative attitudes and beliefs about pain reduce the capacity of patients to cope with painful symptoms, leading to adoption of passive treatment strategies, such as the biomedical model of care that may present a high risk of persistence of pain and disability [46, 47]. In contrast, positive attitudes and beliefs about LBP were argued to be important factors for prevention of pain persistence and disability. The findings of this study supported this claim and showed a protective effect of positive beliefs on chronicity of LBP. Individuals who with positive beliefs about their pain had 33% less chance of reporting chronic LBP, when compared with those with negative beliefs.

In a systematic review of psychological factors predicting chronicity/disability in LBP patients, depressive mood, distress, and somatisation were shown to be associated with the transition of pain from acute presentation to chronic phase [48]. This study also found a strong association between depressive symptomology and chronicity of LBP. The odds of having chronic LBP were higher in individuals who had depressive symptomology and at the borderline for those who had not. For this reason, Pincus et al. [48] indicated the need for the development and testing of clinical interventions targeting these psychological factors in particular.

### Strengths and limitations of the study

The strengths of this study include being a population-based study with a large sample from a socio-economically diverse and representative population. To the best of the authors' knowledge, this is the first ever epidemiological information about the profile of individuals with LBP and factors defining chronicity of pain in Ethiopia. However, the study has some limitations, including possibility of acquiescence response bias, where the respondents agree with the statements regardless of their contents [49]. Such bias may affect validity of the results and lead to wrong conclusions. The literature argues that the extent of occurrence of acquiescence response bias in a survey research ranges from 10 to 20% [50]. In addition, provided the subjective nature of the participants' responses, the addition of alternative scales such as the Visual Analog Scale (VAS) might be helpful for a better understanding of how the individual participants rate their pain. However, this study missed such strategy for improved results. This study may also inherit the limitations of cross-sectional study design, such as the possibility of reverse causality between the reported predicting factors and chronicity of LBP.

## Conclusions

This study provides an overview of the profile of individuals with LBP, including insights into multiple factors associated with chronicity of pain in a representative sample of people in Ethiopia as a sub-Saharan African country. In this population, chronic LBP was found to be a multifactorial biopsychosocial condition with age, educational level, residence, beliefs about pain, and depressive symptomology. Thus, integrating the profile of individuals with LBP and factors associated with chronicity of pain into pain management strategies and future interventions may help to improve patients' outcomes. Future longitudinal studies may also be needed to understand better the true directionality of the association between the identified predicting factors and chronicity of pain and to establish the relationship between chronicity of pain and functionality.

## Supplementary Information

Below is the link to the electronic supplementary material.

Supplementary file1 (DOCX 88 KB)

## Data Availability

The data for this study will be made available in the University of Tasmania data repository.
