# Supplementary material for: Profile of individuals with low back pain and factors defining chronicity of pain: a population-based study in Ethiopia
Source: Qual Life Res. 2022 May 14;31(9):2645–54. doi: 10.1007/s11136-022-03148-5 (PMC9356953; doi:10.1007/s11136-022-03148-5)
Supplement: Supplementary file 1 — Supplementary file1 (DOCX 88 KB) [file 11136_2022_3148_MOESM1_ESM.docx]

**English-language version of the measurement instrument used to collect the data**

1. **Socio-demographic data**
2. Gender  Male  Female
3. Age (in years) __________
4. Ethnicity __________
5. The highest level of education you have completed

No formal education  Technical/Vocational Certificate

Elementary (grade 1-8)  Diploma

Secondary (grade 9-12)  First degree or higher

1. Where do you live?  Urban  Rural
2. Current marital status

Single/never married  Cohabited  Divorced

Married  Separated  Widowed

1. With whom do you live?

Living with nuclear family  Living with nonnuclear family

Living alone

1. How many family members live in your household? _______
2. What is your current occupation, that is, what kind of work do you mainly do? ____________
3. **Pain-interrelated characteristics**
4. In general, how would you rate your LBP on average? *

| 1 | 2 | | 3 | 4 | 5 | 6 | 7 | 8 | 9 | | 10 |
| --- | --- | --- | --- | --- | --- | --- | --- | --- | --- | --- | --- |
| *No pain* | |  | | | | | | | | *Worst imaginable pain* | |

1. How would you rate your health status in the past year?

Excellent  Very good  Good  Fair  Poor

1. How much did LBP interfere with your ability to participate in social activities? *

Not at all  A little bit  Somewhat  Quite a bit  Very much

1. In general, how would you rate your current health status on average?

Excellent  Very good  Good  Fair  Poor

1. How much did LBP interfere with your day-to-day activities? *

Not at all  A little bit  Somewhat  Quite a bit  Very much

1. How long has LBP been an ongoing problem for you? *

Less than one month  Greater than 3 months, but less than one year

1–3 months  1–5 years  More than 5 years

1. **Beliefs about LBP**

To what extent do you agree with the following statements?

1. LBP is not curable

Strongly agree  Agree  Neutral  Disagree  Strongly disagree

1. There is no real treatment for LBP^‡^

Strongly agree  Agree  Neutral  Disagree  Strongly disagree

1. LBP makes everything in life worse^‡^

Strongly agree  Agree  Neutral  Disagree  Strongly disagree

1. Health care providers cannot do anything for LBP^‡^

Strongly agree  Agree  Neutral  Disagree  Strongly disagree

1. LBP eventually stops you from working^‡^

Strongly agree  Agree  Neutral  Disagree  Strongly disagree

1. **Insomnia/sleeping problems**

Please, tick the best option that describes your sleeping in the past year

1. Difficulty falling asleep at night

Never  Seldom  Sometimes  Several times

1. Sleepiness during the day

Never  Seldom  Sometimes  Several times

1. Waking up too early and not getting back to sleep

Never  Seldom  Sometimes  Several times

1. Waking up repeatedly during the night

Never  Seldom  Sometimes  Several times

1. **Depressive symptoms**

Please, tick the best option that describes how you have been feeling in the past year*

1. Hopeless  Never  Seldom  Sometimes  Several times
2. Depressed  Never  Seldom  Sometimes  Several times
3. Worthless  Never  Seldom  Sometimes  Several times
4. Helpless  Never  Seldom  Sometimes  Several times
5. **Health behaviour/lifestyle habits**
6. Khat chewing status

Chewing  Used to chew, but have now quit  Never chewed

- 1. Frequency of chewing khat  Occasionally  Often  Always

1. Cigarette smoking status

Current smoker  Used to smoke, but have now quit  Never smoked

- 1. For how long have you been smoking? _______ years or______ months
  2. Number of cigarettes smoked per day _________

1. Alcohol consumption status  Drinking  Used to drink, but have now quit

Never drunk

- 1. Frequency of alcohol consumption

On regular bases (regular drinker)  Occasionally (social drinker)

1. **LBP associated sequelae**
2. Do have you have another spinal pain in other site(s)?  Yes  No
   1. If yes, at which body part(s)?

Upper back  Neck  Shoulder  Elbow  Hand wrist  Knee  Ankle  Others specify _________

1. Has the pain (LBP) spread down your leg(s)? *  Yes  No
2. In the past one year, have you ever been off work due to your LBP?

Yes  No

- 1. If yes, for how long have you been off work? ________ days or ______ weeks

1. **Health care utilisation data**
2. Have you ever sought consultation or health care from any health care provider for your LBP?  Yes  No

If your answer is 'No' to the above question, please, go to question number 9.

1. Where have you sought consultation or health care for your LBP?

Health post  General hospital

Health centre  Comprehensive specialised hospital

Primary hospital  Private clinic

1. Have you ever had a low back operation?  Yes, one operation  Yes, more than one operation  Never
2. In the past one year, have you sought consultation or healthcare from any health care provider for your current LBP?  Yes  No

If your answer is 'No' to the above question, please, go to question number 9.

1. Where have you sought consultation or healthcare for your LBP?

Health post  General hospital

Health centre  Comprehensive specialised hospital

Primary hospital  Private clinic

1. In the past one year, how many times have you sought consultation or healthcare from the health care provider for your current LBP? ________
2. What type of treatment was prescribed for your LBP?

Injection medicine  Surgery  Back support  Bed rest

Exercise  Massage  Others, specify _____________

1. In the past one year, have you been hospitalised due to your LBP?

Yes  No

- 1. If 'Yes', for how many days have you been stayed in the hospital? __________

1. In the past one year, have you used any traditional medicine for your current LBP?  Yes  No
   1. If 'Yes', please, list the traditional medicines that you have used. _______________

Note:

*-Items taken/adapted from:

Deyo, RA, Dworkin, SF, Amtmann, D, Andersson, G, Borenstein, D, Carragee, E, et al. (2015). Report of NIH task force on research standards for chronic low back pain. Physical Therapy, 95(2), e1-e18. doi:10.2522/ptj.2015.95.2.e1.

‡-Items adapted from:

Bostick, G, Schopflocher, D, Gross, D. (2013). Validity evidence for the back beliefs questionnaire in the general population. European Journal of Pain, 17 (7), 1074-1081. doi:10.1002/j.1532-2149.2012.00275.x.

# Oromo-language Version of the Instrument

Etoophiyaa keessatti wantota sababa dhukkuba dugdaatiif wal'aansa fayyaa fayyadamuu irratti dhiibbaa geessisuu danda'an qorachuuf gaafannoo karaa saayinsaawaa ta'een qophaa'ee mirkanaa'e

1. **Gaafiiwwan hawaasummaa ilaallatan**
2. Koorniyaa  Dhiira  Dhalaa
3. Umrii (waggaan) __________
4. Qomoo __________
5. Sadarkaa barumsaa

Barumsa idilee kan hin baranne

Sadarkaa tokkoffaa (kutaa 1-8)

Sadarkaa 2ffaa (kutaa 9-12)

Tekinikaa fi Ogummaa irraa kan eebbifame/eebbifamte

Dippiloomaa

Digrii jalqabaa ykn isaa ol

1. Bakka jireenyaa  Magaalaa  Baadiyyaa
2. Haala fuudhaa fi heerumaa

Gonkumaa kan hin fuune/hin heerumne

Kan fuudhee/heerumtee waliin jiraachaa jiran

Osoo wal hin fuudhiin kan waliin jiraachaa jiran

Fuudhee/heerumtee kan addaan bahanii garuu seeraan wal hin hiikne

Fuudhee/heerumtee kan wal-hiikan

Kan abbaan manaa irraa du'e/haati manaa jalaa duute

1. Eenyu wajjin jiraatta?

Maatii koo wajjin  Nama biraa kan maatii koo hin taane wajjin

Kophaa koo

1. Mana keessan keessa maatii meeqa taataniit jiraattu? _________
2. Yeroo ammaa kana irra caalaatti hojii maaalii irratti bobbaatee jirta? __________
3. **Wantota dhukkuba dugdaatiin walqabatan**
4. Walumaagalatti, dhukkuba dugdaa kee kana qabxiilee armaan gadii irratti hundaa'uun akkamitti agarsiifta?

| 1 | 2 | | 3 | 4 | 5 | 6 | 7 | 8 | | 9 | 10 |
| --- | --- | --- | --- | --- | --- | --- | --- | --- | --- | --- | --- |
| *Dhukkubbii*  *kan hin qabne* | |  | | | | | | | *Dhukkubbii*  *gar-malee/ hamaa ta'e* | | |

1. Fayyummaa kee waggaa darbee akkamitti ibsita?

Baay'ee baay'ee gaariidha  Baay'ee gaariidha  Gaariidha

Gahaadha  Yaraadha

1. Dhukkubni dugdaa dalagaa hawaasummmaa irratti hirmaannaa kee hagam gufachiise?

Gonkumaa na hin gufachiifne  Baay'ee xiqqoo na gufachiise

Hanga tokko na gufachiise  Xiqqoo ishee na gufachiise  Baay'ee na gufachiise

1. Walumaagalatti, fayyummaa kee yeroo ammaa kana akkamitti ibsita?

Baay'ee baay'ee gaariidha  Baay'ee gaariidha  Gaariidha

Gahaadha  Yaraadha

1. Dhukkubni dugdaa dalagaa idilee kee irraa hagam si gufachiise?

Gonkumaa na hin gufachiifne  Baay'ee xiqqoo na gufachiise

Hanga tokko na gufachiise  Xiqqoo ishee na gufachiise  Baay'ee na gufachiise

1. Dhukkubni dugdaaa erga si qabee hagam sirra ture?

Ji'a tokkoo gadi  Ji'a sadii ol, wagga tokkoo gadi

Ji'a tokkoo hanga ji'a sadii  Wagga tokkoo hanga shanii  Wagga shanii ol

1. **Dhukkuba dugdaa irratti amantaa jiru**

Himoota armaan gadii hagam sirriidha ykn sirrii miti jettee itti amanta?

1. Dhukkuba dugdaa irraa fayyuun hin danda'amu

Sirriidha jedhee baay'een amana  Sirriidha jedheen amana

Sirriidhas sirrii mitis jedhee hin amanu  Sirrii miti jedheen amana

Sirrii miti jedhee baay'een amana

1. Dhukkubni dugdaa wal'aansa hin qabu

Sirriidha jedhee baay'een amana  Sirriidha jedheen amana

Sirriidhas sirrii mitis jedhee hin amanu  Sirrii miti jedheen amana

Sirrii miti jedhee baay'een amana

1. Dhukkubni dugdaa waa'ee jireenyaa hunda mancaasa

Sirriidha jedhee baay'een amana  Sirriidha jedheen amana

Sirriidhas sirrii mitis jedhee hin amanu  Sirrii miti jedheen amana

Sirrii miti jedhee baay'een amana

1. Ogeeyyiin fayyaa dhukkuba dugdaatiif homaa gochuu hin danda'anu

Sirriidha jedhee baay'een amana  Sirriidha jedheen amana

Sirriidhas sirrii mitis jedhee hin amanu  Sirrii miti jedheen amana

Sirrii miti jedhee baay'een amana

1. Dhukkubni dugdaa deemee deemee walumaagalatti hojii nama dhorka

Sirriidha jedhee baay'een amana  Sirriidha jedheen amana

Sirriidhas sirrii mitis jedhee hin amanu  Sirrii miti jedheen amana

Sirrii miti jedhee baay'een amana

1. **Rakkoo hirribaa**

Waggaa darbe keessa, waa'ee hirriba kee ilaalchisee, filannoowwan kennaman keessaa isa sirriitti mudannoo kee ibsu filachuun agarsiisi.

1. Halkan hirriba dhabuu

Gonkumaa na hin mudanne  Darbee darbee na mudataa ture

Yeroo tokko tokko na mudataa ture  Yeroo baay'ee na mudataa ture

1. Yeroo guyyaa hirribni si qabuu

Gonkumaa na hin mudanne  Darbee darbee na mudataa ture

Yeroo tokko tokko na mudataa ture  Yeroo baay'ee na mudataa ture

1. Barii barraaqa hirriba irraa dammaqxee hirribni deebi'ee si qabuu dhiisuu

Gonkumaa na hin mudanne  Darbee darbee na mudataa ture

Yeroo tokko tokko na mudataa ture  Yeroo baay'ee na mudataa ture

1. Halkan hirriba keessaa dadammaquu

Gonkumaa na hin mudanne  Darbee darbee na mudataa ture

Yeroo tokko tokko na mudataa ture  Yeroo baay'ee na mudataa ture

1. **Mallattoowwan mukaa'uu**

Tokkoon tokkoo qabxiilee armaan gadii irratti, waggaa darbe keessa hagam akka sitti dhaga'amaa ture filannoowwan kennaman keessaa isa sirriitti ibsu filachuun agarsiisi

1. Abdi-dhabeessa/abdi-dhabeettii

Gonkumaa natti hin dhaga'amne

Darbee darbee natti dhaga'amaa ture

Yeroo tokko tokko natti dhaga'amaa ture

Yeroo baay'ee natti dhaga'amaa ture

1. Mukaa'uu

Gonkumaa natti hin dhaga'amne

Darbee darbee natti dhaga'amaa ture

Yeroo tokko tokko natti dhaga'amaa ture

Yeroo baay'ee natti dhaga'amaa ture

1. Gati-dhabeessa/Gati-dhabeettii

Gonkumaa natti hin dhaga'amne

Darbee darbee natti dhaga'amaa ture

Yeroo tokko tokko natti dhaga'amaa ture

Yeroo baay'ee natti dhaga'amaa ture

1. Gargaarsa-dhabeessa/Gargaarsa-dhabeettii

Gonkumaa natti hin dhaga'amne

Darbee darbee natti dhaga'amaa ture

Yeroo tokko tokko natti dhaga'amaa ture

Yeroo baay'ee natti dhaga'amaa ture

1. **Araada fayyaan walqabatan/haala jireenyaa**
2. Jimaa qama'uu

Nan qama'a  Qama'aan ture, amma garuu dhiiseen jira

Gonkumaa qama'ee hin beeku

- 1. Ni qamaata yoo ta'e, Jimaa yeroo hammamiitti qamaata?

Darbee darbee  Yeroo baay'ee  Yeroo mara

1. Tamboo xuuxuu

Nan xuuxa  Xuuxaan ture, amma garuu dhiiseen jira

Gonkumaa xuuxee hin beeku

- 1. Ni xuuxxa yoo ta'e, erga xuuxuu jalqabdee waggaa/ji'a meeqa ta'e?

wagga _______ ykn ji'a _______

- 1. Ni xuuxxa yoo ta'e, guyyaatti sijaaraa meeqa xuuxxa? __________

1. Dhugaatii alkoolii

Nan dhuga  Dhugaan ture, amma garuu dhiiseen jira

Gonkumaa dhugee hin beeku

- 1. Ni dhugda yoo ta'e, yeroo hammamiitti dhugda?

Yeroo mara  Darbee darbee

1. **Miidhaa dhukkuba dugdaatiin walqabatan**
2. Dhukkuba dugugguruu bakka biraa irraa qabdaa?  Eeyyee  Lakki
   1. Ni qabda yoo ta'e, bakka kam irraa?

Dugda gara ol-aanu  Morma  Gateettii  Ciqilee

Burrisa harkaa  Jilba  Burrisa miilaa (koroonyoo)

Bakka biroo, caqasi _________

1. Dhukkubbiin dugdaa kee kun gara miilaatti gadi faffaaca'eeraa?

Eeyyee  Lakki

1. Wagggaa darbe keessa, sababa dhukkuba dugdaatiin hojii irraa haftee beektaa?

Eeyyee  Lakki

- 1. Gaaffii armaan olii 'Eeyyee' jechuun deebifte yoo ta'e, yeroo hammamiif hojii irraa hafte? Guyyaa ______ ykn torban ______

1. **Gaafiiwwan wal'aansa fayyaa fayyadamuu ilaallatan**
2. Dhukkuba gugdaa keetiif takkaa gorsa yookiin wal'aansa ogeessa fayyaa argattee beektaa?  Eeyyee  Lakki

Gaaffii armaan olii 'Lakki' jechuun deebifte yoo ta'e, gara gaaffii lakkoofsa 9 tti ce'i.

1. Gorsa yookiin wal'aansa ogeessa fayyaa eessaa argatte?

Kellaa fayyaa  Hospitaala waliigalaa

Buufata fayyaa  Hospitaala tajaajila addaa

Hospitaala jalqabaa  Kilinika dhuunfaa

1. Dugda kee irra waldhaansi baqaqsanii yaaluu takkaa siif godhamee beekaa?

Eeyyee  Lakki

1. Waggaa darbe keessa, dhukkuba dugdaa kee isa ammaa kanaaf gorsa yookiin wal'aansa ogeessa fayyaa argattee beektaa?  Eeyyee  Lakki

Gaaffii armaan olii 'Lakki' jechuun deebifte yoo ta'e, gara gaaffii lakkoofsa 9 tti ce'i.

1. Gorsa yookiin waldhaansa ogeessa fayyaa eessaa argatte?

Kellaa fayyaa  Hospitaala waliigalaa

Buufata fayyaa  Hospitaala tajaajila addaa

Hospitaala jalqabaa  Kilinika dhuunfaa

1. Waggaa darbe keessa, gorsa yookiin waldhaansa ogeessa fayyaa al meeqa argatte? __________
2. Dhukkuba dugda keetiif qoricha/waldhaansa akaakuu kamiitu siif ajajame/kenname?

Qoricha lilmeen kennamu  Sochii qaamaa

Waldhaansa baqaqsanii yaaluu  Sukkuummii/dhidhiibbaa

Utubbii dugdaa  Kan biroo, caqasi _____

Boqonnaa siree irraa

1. Waggaa darbe keessa, sababa dhukkuba dugdaa keetiif hospitaalaa galtee ciiftee beektaa?  Eeyyee  Lakki
   1. Gaaffii armaan olii 'Eeyyee” jechuun deebifte yoo ta'e, guyyaa meeqaaf hospitaala ciifte? __________
2. Waggaa darbe keessa, dhukkuba dugdaa kee isa ammaa kanaaf wal'aansa ykn qoricha aadaa fayyadamtee beektaa?  Eeyyee  Lakki
   1. Gaaffii armaan olii 'Eeyyee' jechuun deebifte yoo ta'e, wal'aansa ykn qoricha aadaa fayyadamte eeri ___________________
